# Shoreline dynamics in response to sea-level changes on the eastern coast of Bangladesh

**DOI:** 10.1007/s10661-025-14375-x

**Published:** 2025-07-23

**Authors:** Md.Mehedi Hasan Saddam, Khandaker Tanvir Hossain, Porni Mollick, Md. Salauddin, Sanjoy Roy, Arif Uddin Ahmad, Ratan Chandra Bhowmick, Md.Ashikul Islam Rony

**Affiliations:** 1 Disaster and Climate Risk Management Department, Bangladesh Red Crescent Society (BDRCS), Dhaka, Bangladesh; 2 Department of Geography and Environment, Jagannath University, Dhaka, 1000 Bangladesh; 3 Faculty of Science and Technology, Research Institute for the Environment and Livelihoods, Charles Darwin University, Darwin, Australia; 4 Food and Agriculture Organization of the United Nations, Dhaka, Bangladesh; 5 Bengal Institute- Architecture, Landscapes and Settlements, Dhaka, Bangladesh; 6 Department of Geo-Information Science and Earth Observation, Patuakhali Science and Technology University, Patuakhali, Bangladesh; 7 Faculty of Bio-Science and Engineering, Vrije Universiteit Brussel, Brussels, Belgium; 8 Bangladesh University of Professionals, Dhaka, Bangladesh; 9 Landscape Analysis and Management Laboratory, Department of Geography, University of Girona, Girona, Spain

**Keywords:** Shoreline change, Sea-level rise, River systems, Coastal geomorphology, Mangroves, Remote sensing

## Abstract

The eastern coast of Bangladesh has undergone significant geomorphic changes since 1990, with the shoreline predominantly prograding towards the Bay of Bengal. Analysis indicates that the northeastern (Mirsarai- Banshkhali) coast experienced seaward progradation, with a maximum progradation of 3.87 km and an average rate of 0.13 km/year over the past 30 years. Conversely, the south-eastern coast (Cox’s Bazar toward Teknaf) exhibited landward movement due to erosion, with a maximum retreat of 2.9 km and an average rate of 0.096 km/year. These changes were quantified using remote sensing and the Digital Shoreline Analysis System (DSAS), an ArcGIS extension, to calculate Net Shoreline Movement (NSM) and Linear Regression Rate (LRR) with 95% confidence intervals. Shorelines were manually digitized from Landsat images at a fixed scale of 1:2500 to ensure precision. Mean Sea Level (MSL) data from two tidal stations revealed fluctuating trends over time. At Enayethat station, a weak uphill positive correlation was found between sea-level rise and shoreline erosion (*R*^2^ = 0.06), whereas at Lemshikhali station, the MSL decreased between 1990 and 2019, and a moderate uphill positive correlation (*R*^2^ = 0.43) with shoreline erosion was observed. The findings indicate that accretion along the eastern shoreline surpasses erosion. Sedimentation in the estuary and the stabilizing influence of eastern hills contribute to a long-term trend of shoreline stability and seaward expansion. Importantly, no direct relationship was identified between sea-level rise and coastal erosion, highlighting the dominance of localized geomorphic and sedimentary processes in shaping shoreline dynamics. These results could reflect similar patterns of change in other tropical shorelines worldwide.

## Introduction

The shoreline is a dynamic, transitional, and sensitive zone which is often influenced by both natural and anthropogenic activities. It not only distinguishes the sea and land boundary but also retains the coastal environment. Shorelines frequently change in both the short-term and long-term due to hydrodynamic and geomorphological variations (Klemas, 2013). Regular and precise delineation and monitoring of the shoreline can help us to understand the coastal processes. It is necessary for coastal zone management, sediment budgets, hazard zone identification, and erosion-accretion studies (Masselink et al., 2014). Shoreline shifting readily affects the resources of intertidal areas. Moreover, this movement may cause a disparity in the varieties of resources and the ecological system in coastal areas that may influence the survival and development of coastal people (Tao, 2006). Sea-level rise threatens the low-lying coastal regions where the population concentration is very high (Thompson et al., 2016). Shoreline change due to the rising sea level has become an important issue that has displaced a vast amount of the coastal population. Climatic factors are vastly influenced by the increase in the height of sea waves, storm surge, wind speed and directions, and coastal flooding in recent decades (Gargiulo et al., 2020). IPCC has reported that the recent air temperature is increasing faster than the previous centuries (Thornes, 2002), and the average sea level has risen by 3.2 mm/year (IPCC, 2014) which may lead to the shifting of shoreline radically. According to a special report by IPCC (2019), the global mean sea level has increased to 21 cm from 1902 to 2015 and has the potential to reach 43–84 cm by the twenty-first century (Masson-Delmotte et al., 2019; McMichael et al., 2020). Two different tide gauges in the southeastern coastal region of Bangladesh recorded that the sea level has risen (Brammer, 2014). Bangladesh is a lower riparian country that is hydrologically and morphologically dominated by the Ganges Brahmaputra Meghna (GBM) river system and the Bay of Bengal (Ahmad, 2019) where the siltation process is active in estuaries (Kuehl et al., 2005; Rahman et al., 2018; Rashid & Rashid, 2014). Recent studies revealed that Bangladesh is one of the most climate-vulnerable countries in the world due to sea-level rise (Uddin et al., 2019), where the intensity and frequency of cyclones and storm surges are very high (IPCC, 2014; Saenger & Siddiqi, 1993). Climate change impacts and frequently occurring cyclones and storm surges, along with anthropogenic activities, have made the coastal zones more unstable, resulting in the loss of coastal resources for low-lying overpopulated countries like Bangladesh (Haque et al., 2020).

Delineation and measurement of coastline shifting are most significant for understanding the coastal geomorphological process. It helps to investigate the relationship between sea level change and shoreline movement and assists coastal zone management, coastal flood prediction, and erosion-accretion studies. Conventional techniques of direct field survey to delineate the shoreline are nearly impossible, which can be accurately done by aerial photographs or satellite images. The Digital Shoreline Analysis System (DSAS), an extension of ArcMap, can identify the coastline change accurately. Shoreline change has been quantified globally using the Digital Shoreline Analysis System (DSAS) (Sarwar & Woodroffe, 2013; Chettiyam Thodi et al., 2023). This tool incorporates several widely used statistical models for shoreline change analysis, including Net Shoreline Movement (NSM), End Point Rate (EPR), and Linear Regression Rate (LRR) (Gopinath et al., 2023; Himmelstoss et al., 2018; Nassar et al., 2019; Salghuna & Bharathvaj, 2015). These techniques are more effective due to their cost-effectiveness and lower manual error compared to other techniques (Thieler & Danforth, 1994). Recently, satellite data have been widely used for assessing the changes in delta and coastal regions. Abou Samra and Ali (2021) applied satellite data and DSAS tools to detect coastal changes along the Nile Delta, Egypt, between 1984 and 2018. An ISODATA classification method was applied to evaluate the erosion and accretion pattern. Navera and Ahmed (2021) utilized remote sensing data and NDVI techniques to assess the shoreline change along the Cox’s Bazar and Teknaf coast. Anwar et al. (2022) used remote sensing, sea level, and meteorological data for morphological change analysis of the eastern coast (Kutubdia Island and Cox’s Bazar District). The shoreline was delineated based on reflectance intensity variations and edge detection methods. Abdullah et al. (2019) used satellite data for spatiotemporal dynamics of new land development on the Bangladesh coast from 1996 to 2015. The study revealed that from 1996 to 2015, Bangladesh’s coast gained a net of 78.79 km^2^ of land. They used random forest and support vector machine classifications for the delineation of the boundary of land and water.

Most of the previous studies used band ratios like NDVI, NDWI, MNDWI, image classification techniques, histogram equalization (Abdullah et al., 2019; Abou Samra & Ali, 2021; Alesheikh et al., 2007; Anwar et al., 2022; Cassé et al., 2012; Navera & Ahmed, 2021; Toure et al., 2019), and adaptive threshold (Aedla et al., 2015) techniques to delineate the land–water boundary using satellite imageries. But in open water like coastal areas, it is quite tough to detect the coastline accurately through conventional band ratio and threshold techniques because of tidal fluctuation, the coarser pixel size (30- to 60-m spatial resolution) of satellite data, and similarities of spectral reflectance (Kumar et al., 2010). Therefore, it is essential to extract land pixels (high water line) through visual interpretation to obtain the coastline precisely in a dynamic coastal environment (Dewan et al., 2017). Only a few studies used on-screen manual digitization in different scales 1:250,000, 1:50,000, and 1:5000, respectively (Dewan et al., 2017; Gupta et al., 2013; Hossain et al., 2013; Kankara et al., 2015; Yadav et al., 2021). In the present study, multi-temporal Landsat imagery from 1990 to 2020 was utilized for shoreline delineation. All images were interpreted at a fixed scale of 1:2500 to ensure consistent visual resolution and accurate identification of the land–water interface. To enhance visual contrast between terrestrial and aquatic features, different false-color band combinations were applied. Specifically, RGB 7–5-1 was used for Landsat TM/ETM + sensors, while RGB 7–5-3 was employed for Landsat OLI imagery. These combinations effectively highlighted the boundary between land and water during the digitization process. To understand the influences of sea level on the coastline, sea level data were collected from one nearby tide gauge station due to the unavailability of a tidal gauge station along the study area. Finally, a correlation was built up to know the impact of sea level change on erosion and accretion along the entire study area while three primary objectives of the study were marked: (i) to calculate the net shoreline shifting and the rate of shifting in the last 30 years, (ii) to measure the erosion and accretion of the coastal area, and (iii) to investigate the relationship between MSL and erosion-accretion. These primary objectives helped to examine the impact of sea level changes on shoreline shifting.

This research was conducted on the eastern coastline of Bangladesh, which holds substantial significance for the national economy due to its diverse contributions across sectors such as tourism, fisheries, salt production, employment generation, energy development, and international trade (Ahamed et al., 2020). This region is home to Cox’s Bazar, the world’s longest uninterrupted sandy beach, which faces increasing vulnerability from projected sea-level rise (Anwar et al., 2022). Currently, two major infrastructure initiatives, such as the Matarbari Deep Sea Port and the Matarbari Coal-Fired Power Plant, are underway in the central coastal zone, aiming to bolster economic development by enhancing the efficiency of maritime trade (Tareq et al., 2021). Furthermore, the Chittagong Sea Port, also located along this coastline, plays an important role in both national and international trade logistics, accounting for more than 90% of the country’s seaborne trade volume (Saha, 2021). Considering the region’s high economic value and infrastructure density, it is imperative to evaluate the potential impacts of sea-level rise on the eastern coast (Uzzaman, 2014). Such an assessment is essential for informing policy decisions and developing targeted adaptation and mitigation strategies (Ortiz, 1994). Thus, we selected this study area based on its critical economic relevance and susceptibility to climate-induced coastal hazards.

## Materials and methods

### Study area

**Fig. 1:**
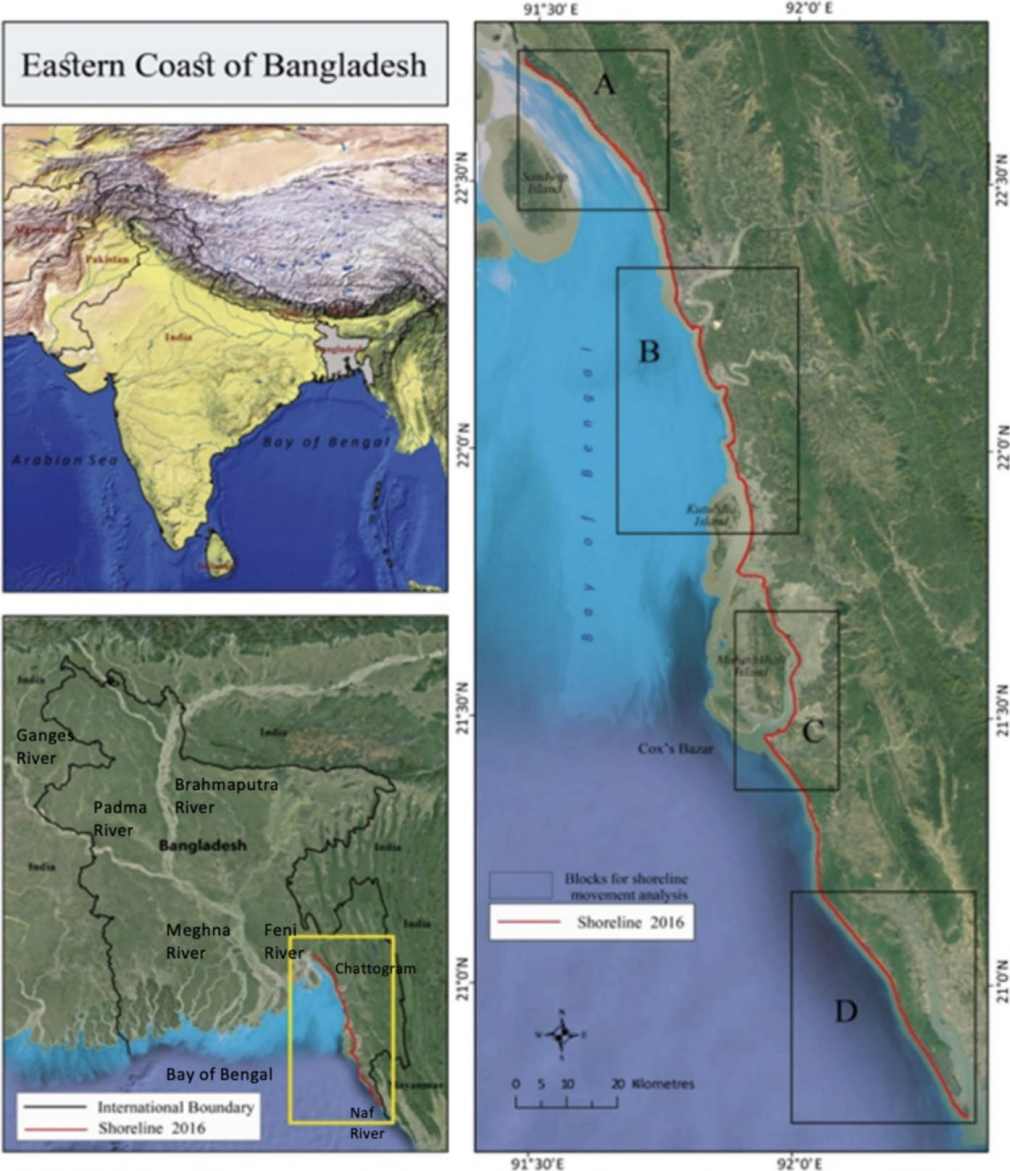
The geographical settings of the study area; Source: ESRI Basemap (National Geographic Style) 2016. The yellow block in the Bangladesh map shows the exact boundary of the current study, which is segmented into four different blocks, namely A, B, C, and D, that are placed with dark boxes. The shoreline position of 2016 is visualized in red color and digitized by the Authors (2016)

Bangladesh has an estimated coastline of approximately 710 km (Sarwar & Khan, 2007) of which the present study focuses on a 32% segment. The study area extends from the northern boundary in Chattogram District to the southern boundary in Cox’s Bazar District. Geographically, the eastern coastal zone begins at Bodor Mokam, located at the southern tip of the mainland near the Feni River estuary. This region is characterized by a series of small hills that run parallel to the coastline. Several major rivers, including the Karnafuli, Sangu, and Matamuhury, discharge into the Bay of Bengal within this zone. Additionally, the Naf River marks the international boundary between Bangladesh and Myanmar as it flows into the Bay of Bengal (Fig. 1).

Soil characteristics of the eastern coastal zone are dominated by submerged sands and mudflats. The submerged sand of the zone has formed a long sandy beach of 145 km from Cox’s Bazar towards Teknaf. The topography of the eastern coast is more diverse than other regions of the country. High north–south striking hill ranges occupy Chattogram, Cox’s Bazar and three hill tracts districts. The hill becomes higher towards the east, reaching a maximum height of 1003 m. The lowest range generally follows the eastern Bay of Bengal from the Feni River to the Naf River and continues southwards across the Myanmar border.

### Geospatial and statistical analysis

**Fig. 2:**
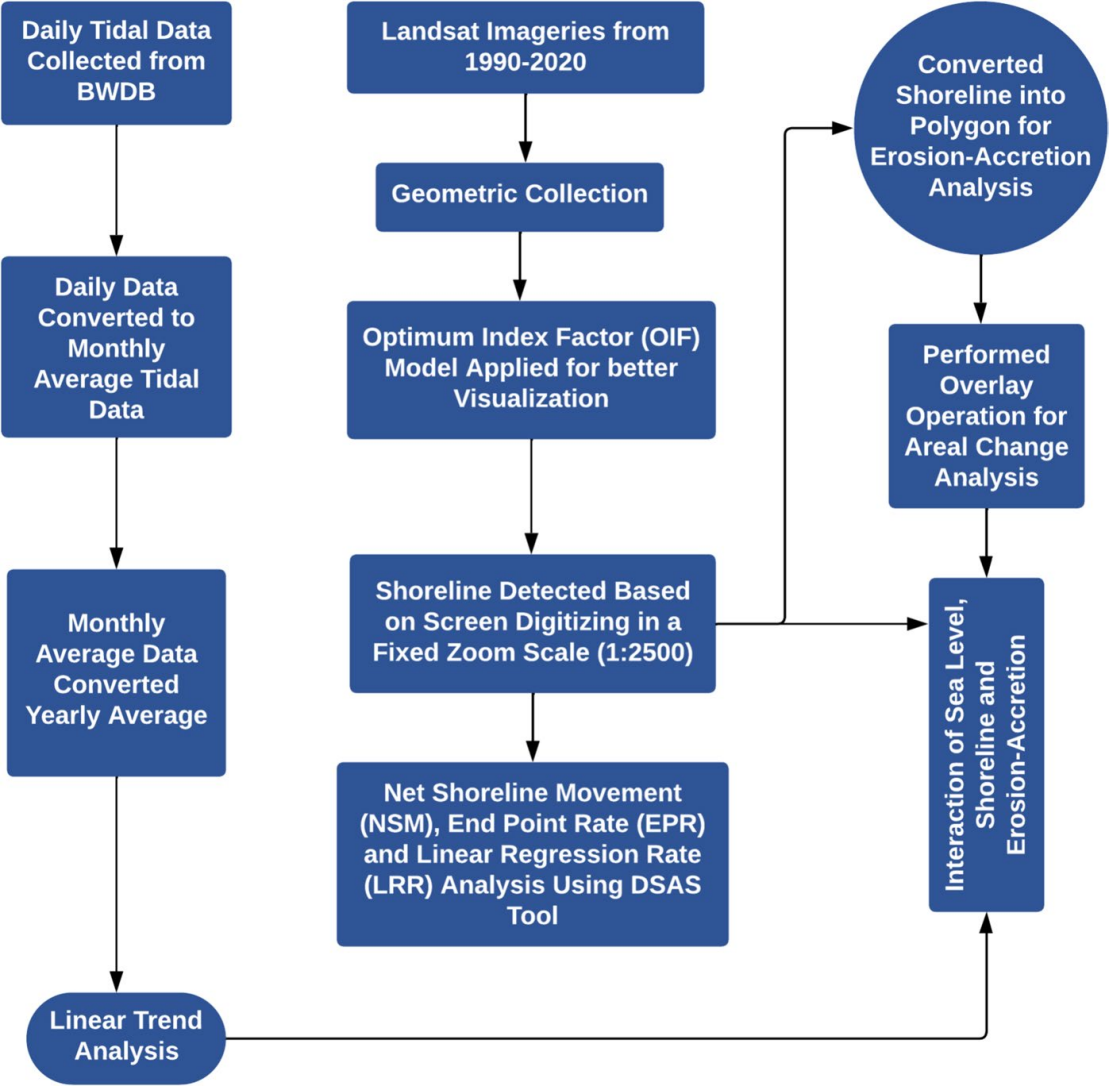
A detailed theoretical framework of this study where the total procedure is addressed

To assess the coastline shifting and measure the sea level changes, the study was conducted into three steps: (i) interpretation of satellite (Landsat) imageries for coastline delineation, (ii) statistical analysis to calculate the rate of coastline shifting and erosion-accretion rate along the coast, and (iii) performing time series analysis of sea level data to understand the sea level changes over time, and finally, linear regression analysis to understand the impact of sea level changes on coastline shifting. Figure 2 depicts the theoretical framework of the study.

#### Image processing

This study employed multi-temporal satellite imagery from the Landsat archive, originally acquired at a spatial resolution of 30 m and referenced to the WGS 84/UTM Zone 46 N coordinate system. Specifically, Landsat 5 TM images were acquired on 31 December 1990 and 31 December 2000, while Landsat 8 OLI images were acquired on 31 December 2010 and 31 December 2020 during rising tide conditions. Several images were downloaded randomly from October to February and then cross-checked with high tide data. Only those images that were well visible with high tide data were selected in the present study. Cloud cover of less than 5% has been chosen for coastline delineation. Three image processing platforms (ENVI, ILWIS, and ERDAS IMAGINE) were deployed for image analysis. These were used simultaneously to get the best performance during digitization. Digitization of coastlines was performed manually at a fixed zoom (1:2500) by a single operator in ArcGIS. This is the most strengthened point of this study, while other previous studies Yadav et al. (2021) and Dewan et al. (2017) used a 5000 to 250,000 scale during manual digitization. Different band combinations (RGB 7–5-3, RGB 4–3-2) were applied that were derived from the ILWIS software, which helped to identify the edge of land and water more precisely. All the acquired images were geometrically and radiometrically corrected to remove the atmospheric and topographical influences. Spectral information of optical images is significantly influenced by atmospheric elements (i.e., water content, dust particles, aerosols, clouds, and varying sun angles) (King et al., 1992). Auto Sync extension of ERDAS IMAGINE was used for image referencing where RMSE less than 5 m was considered and the Dark Object Subtraction (DOS) method of ENVI for atmospheric correction, which was used for calculating Optimum Index Factor (OIF) values by ILWIS (ensure best statistical choice to create a better combination of satellite images) and OIF ensured the band combination for better coastline visualization. In some cases, OIF did not perfectly match for better visualization; it was manually adjusted for the digitization of the coastline.

#### Extraction of the shoreline

The shoreline was visually extracted by a single operator (Dewan et al., 2017; Yadav et al., 2021). OIF values from ILWIS gave better band combination choices. But in some cases, the operator changed the band composition, and it did not work better, but in all cases, the zoom scale was fixed at 1:2500. This fixed zoom scale makes the work stronger and more unique than other studies (Dewan et al., 2017; Gupta et al., 2013; Hossain et al., 2013; Kankara et al., 2015; Yadav et al., 2021). There are no unique techniques available to detect shorelines due to the diversified characteristics of the coast (Ma et al., 2008; Sun et al., 2011). In this study, the landward margin of the mangrove fringes was considered as the shoreline boundary, as appeared in the Landsat images (Mishra et al., 2019; Xu et al., 2014).

#### Extraction process for MSL

Mean Sea Level (MSL) is defined as the seawater level from a land benchmark over an average period, either monthly or annually. In Bangladesh, where the siltation process is active, obtaining long-term continuous tidal data from a single gauge station is often challenging. To prevent biases and overestimation of data, similar high-tide and low-tide data were manually identified and edited. The tidal data period was shorter than the image data.

To assess the impact of sea level change, along with erosion and accretion, two tidal stations from the Bangladesh Water Development Board (BWDB) were used in this study: Enayethat station (near Chattogram district) from 1989 to 2019, and Lemesikhali station (near Cox’s Bazar district) from 1990 to 2019. The daily tidal data were compiled into monthly data, and the monthly averages were further compiled into yearly tidal data.

### Calculation of shoreline changes

DSAS version 5.1, an extension for ArcMap 10.0, was used to calculate the Net Shoreline Movement (NSM) and Linear Regression Rate (LRR), incorporating 95% confidence intervals to assess shoreline change trends with statistical significance. For calculating the change rate of the shoreline by establishing a baseline and shoreline (Himmelstoss et al., 2018; Thinh & Hens, 2017), NSM and LRR quantified and analyzed variations in shoreline positions both in spatial and temporal scales. NSM represented the distance between the earliest and most recent shoreline positions, providing a straightforward measure of shoreline advance or retreat over time. It is particularly useful for identifying areas of significant erosion or accretion. In contrast, LRR calculated the rate of shoreline change by fitting a least-squares regression line to multiple shoreline positions over time, yielding a more robust and statistically reliable estimate of change, especially in the presence of variable shoreline movement (Himmelstoss et al., 2018; Thinh & Hens, 2017). So, NSM measures the total distance between the youngest and the oldest coastline in every transect in m units, while LRR is an approach for estimating the rate of erosion and accretion in the long term (Crowell et al., 1997; Thinh & Hens, 2017). As the eastern coast is discontinuous, a reference baseline was created on land while the coastline was in the offshore direction. To measure the shoreline change and average and annual change rate (landward/seaward), transect lines were created at 100-m intervals. In the case of discontinuous shorelines (the shoreline was interrupted by the estuary of the river), a few transects were removed. For calculating the real change along the coastal areas, the geoprocessing tool intersect was used, where the overlay operation was performed. There were 2522 transect lines constructed using a 5000-m buffer zone on the landward margin, among which a few were deleted for the discontinuation of the shoreline, and finally, 1479 transects were grouped arbitrarily into four segments based on the shoreline movement pattern.

### Validation and accuracy assessment

The accuracy assessment and validation for the shoreline change analysis were conducted using the radiometric corrections that compared classified shoreline change data with reference data (geopoints) collected from 2020’s fieldwork. Radiometric corrections, including the Dark Object Subtraction (DOS) method and linear enhancement techniques, were applied to improve the quality and consistency of the multi-temporal satellite imagery. Geometric rectification was performed using the *ERDAS Imagine AutoSync* workstation, which employed the Automatic Point Matching (APM) algorithm. The geometric alignment of the images achieved a sub-pixel level accuracy, with a mean registration error of less than 0.6 m per pixel. A third-order polynomial transformation was used for image rectification, ensuring that the percentage of geometric error remained below 3% per pixel. The accuracy of the rectification process was further validated using root mean square error (RMSE) calculations across the selected timeframes. The RMSE values were 0.372 for the year 2000, 0.567 for 2010, and 0.530 for 2020, using the 1990 imagery as the base year. These low RMSE values confirm a high degree of positional accuracy, validating the robustness of the geometric correction process applied to the Landsat datasets used in this study.

## Results

### Rate of shoreline change

**Fig. 3:**
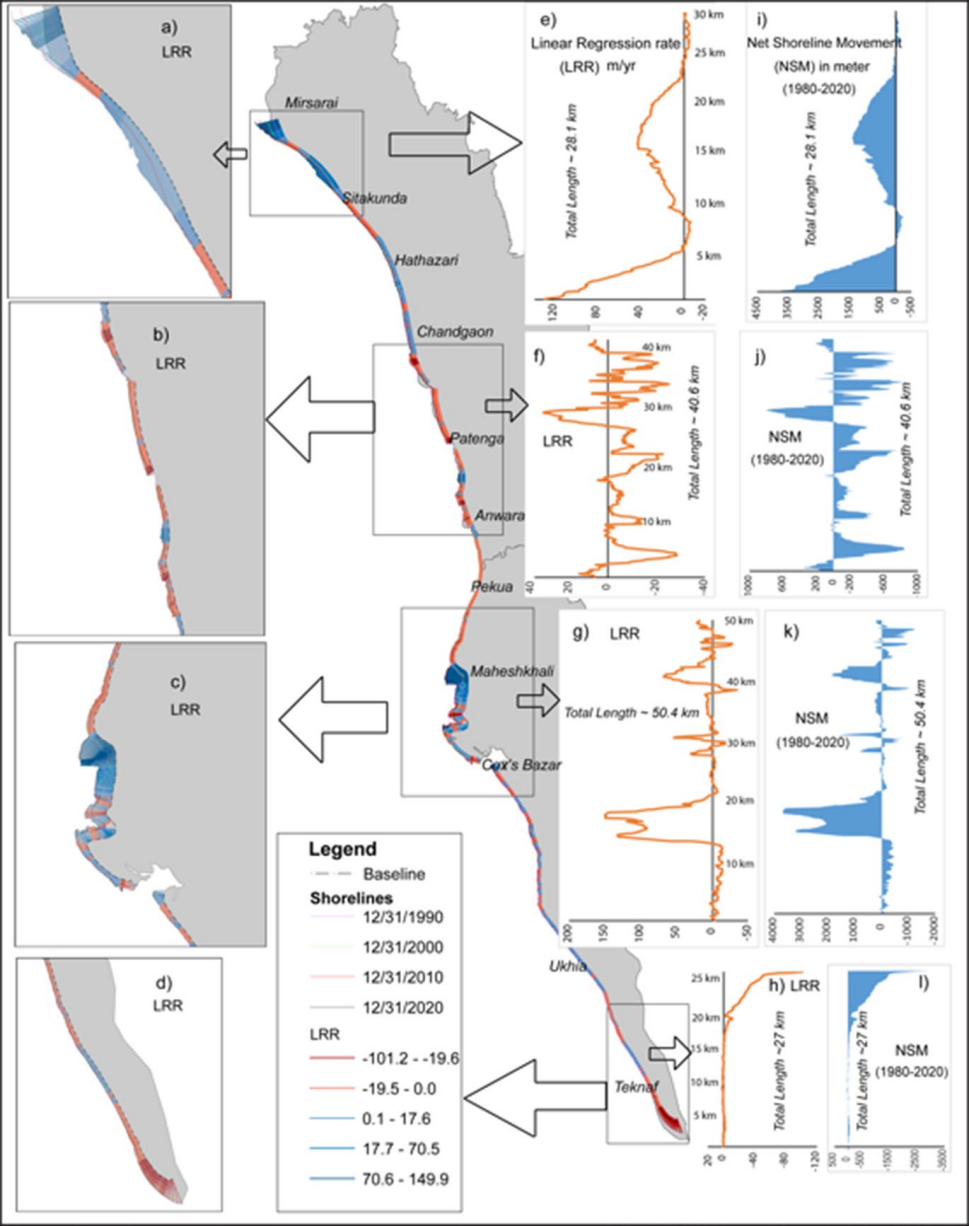
Representation of the Linear Regression Rate (LRR) and Net Shoreline Movement (NSM) of four segments along the study area from the period 1990 to 2020

The change rate of the shorelines derived from DSAS is addressed in Table 2. Segment A, the geographical extent is from Mirsarai toward Sitakunda sea beach, consists of 288 transects, of which 218 transects moved towards the sea (positive movement), where the highest movement was recorded at 3.87 km, and the rest of the transects showed landward movement (negative movement) (Table 2, Fig. 3). In this segment, 75% of transect movement experienced positive movement (seaward while only 25% showed the landward movement of the shoreline. The minimum shoreline movement recorded is − 398.8 m in this segment (Table 2), where a far difference was noticed from the mean shoreline change (636.26 m). The calculated result of LRR experienced differences in changing rates of shoreline. The mean LRR of segments A, B, C, and D are 22.0 m/year, − 4.7 m/year, 15.59 m/year, and − 8.5 m/year, respectively (Table 1, Fig. 3). Segment A, experienced the average highest mean accretion of 22.0 m/year compared to other segments. The overall result expresses the accretion trend of the shoreline in this segment. In segment B, a total of 418 transects were drawn by DSAS. This segment extended from the estuary of the Karnafuli River to the upper stream of the Maheskhali channel. By analyzing the result, approximately 76% of transects showed erosion value while only 24% of transects experienced accretion value. On average, the Net Shoreline Movement (NSM) was − 186.8 m which meant the landward movement of the shoreline was more dominant in this section while the maximum and minimum movement of the shoreline attained 797.9 m and − 849.7 m respectively (Table 2, Fig. 3). Segment C was extended from along the Maheshkhali Island towards the longest sea beach in the world Cox’s Bazar, having a total of 503 transects, among which there are approximately 54% of transects experienced landward movement of the shoreline while 46% experienced accretion (Table 2, Fig. 3). A previous study depicted that accretion was more dominating along the Cox’s Bazar shoreline, and the rate of shifting towards the sea was 3.91 mm/year from 1989 to 2016 (Anwar et al., 2022). Another study that was conducted along the Maheshkhali Island, an adjacent island of Cox’s Bazar, illustrates that the shoreline has moved toward the sea at a rate of 11.85 mm/year in the recent three decades (Saddam et al., 2023). In our present study, we found that the maximum movement of the shoreline was 3871.4 m from the period 1990 to 2020 (30 years) with an average rate of 40 m/year. In segment D, a total of 270 transects were analyzed. Among these, 202 transects experienced erosion, while only 68 transects showed accretion. The landward movement of the shoreline was dominant in this segment when compared to all other segments. The shoreline was eroding at an average rate of 72.46 m/year, while it accreted at an average rate of only 1.35 m/year. The average LRR of this segment was − 8.15 m/year. The highest seaward movement rate was 96.79 m/year in segment A, while the highest landward movement was recorded (− 72.46 m/year) in segment D (Table 1 and Fig. 3).

**Table 1:** The quantification of shoreline changes in four different segments of the eastern coast in Bangladesh during the period from 1990 to 2020

Descriptive statistics	Segment			
	**A**	**B**	**C**	**D**
Total number of transects	288	418	503	270
Number of positive transects	218	100	233	68
Number of negative transects	70	318	270	202
Maximum shoreline change (m)	3871.4	797.9	3690.41	53.74
Minimum shoreline change (m)	− 398.8	− 849.73	− 1235.19	− 2898.32
Mean shoreline change (m)	636.26	− 168.08	293.659	− 244.67
Maximum LRR	131.07	26.22	149.89	1.83
Minimum LRR	− 6.03	− 28.3	− 33.94	− 101.2
Mean LRR	22.0	− 4.7	15.59	− 8.15
Total erosion (hectare)	59.063	978.349	707.057	1006.092
Total accretion (hectare)	2780.33	206.211	1807.36	9.4

Although the total number of positive transects (619) is lower than the number of negative transects (860), the rate of movement and net shoreline change are more significant among the positive transects. This indicates that despite fewer instances of accretion, the magnitude of shoreline advancement is greater than that of erosion. The corresponding spatial analysis further supports this pattern, with the total accreted area (4803.301 ha) exceeding the eroded area (2750.561 ha), demonstrating that accretion is the dominant process along the shoreline during the study period (Table 1; Fig. 3).

### Erosion-accretion along the shoreline of the eastern coast of Bangladesh

**Fig. 4:**
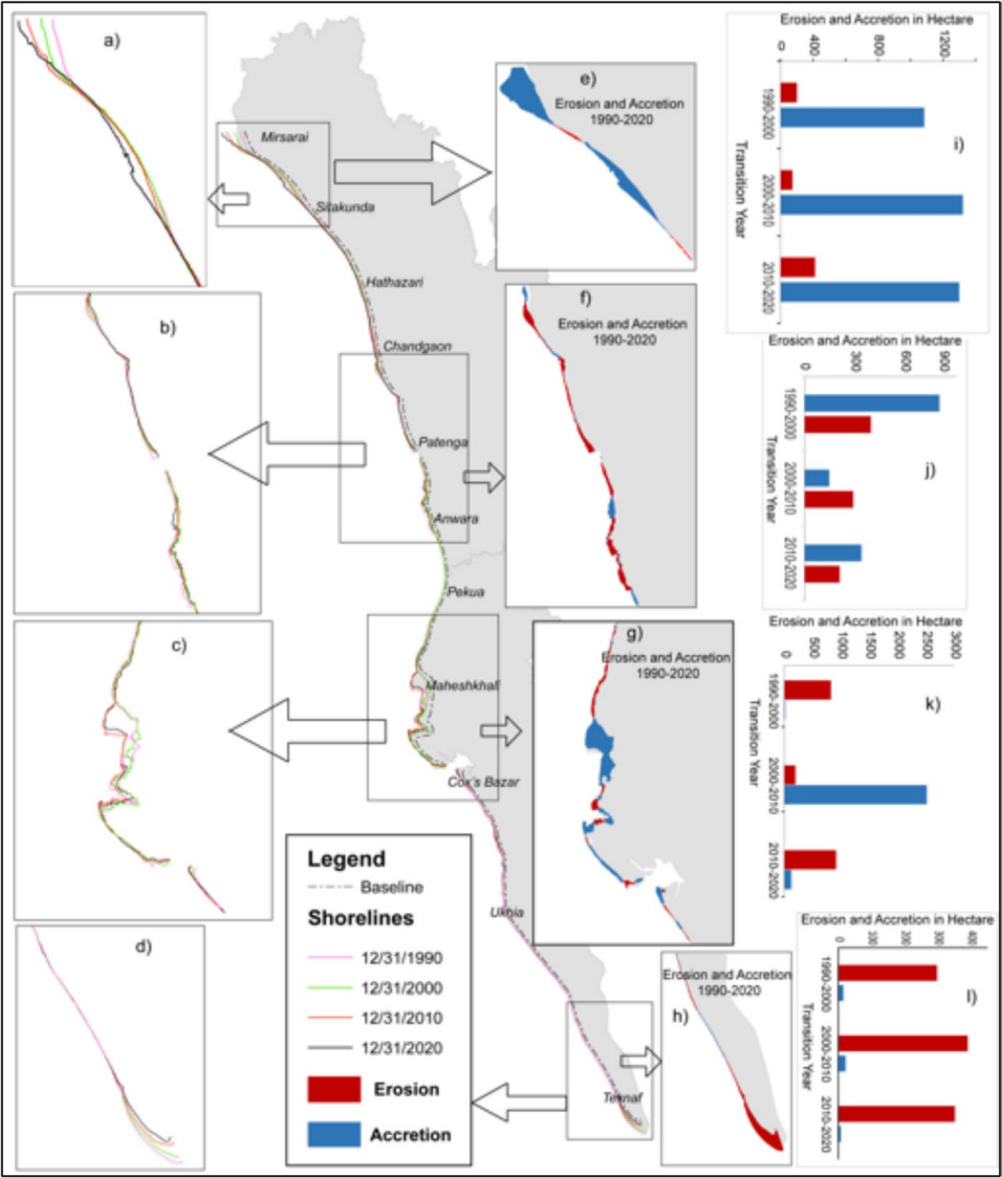
The dynamics of shoreline changes along the eastern coast of Bangladesh from 1990 to 2020

**Fig. 5:**
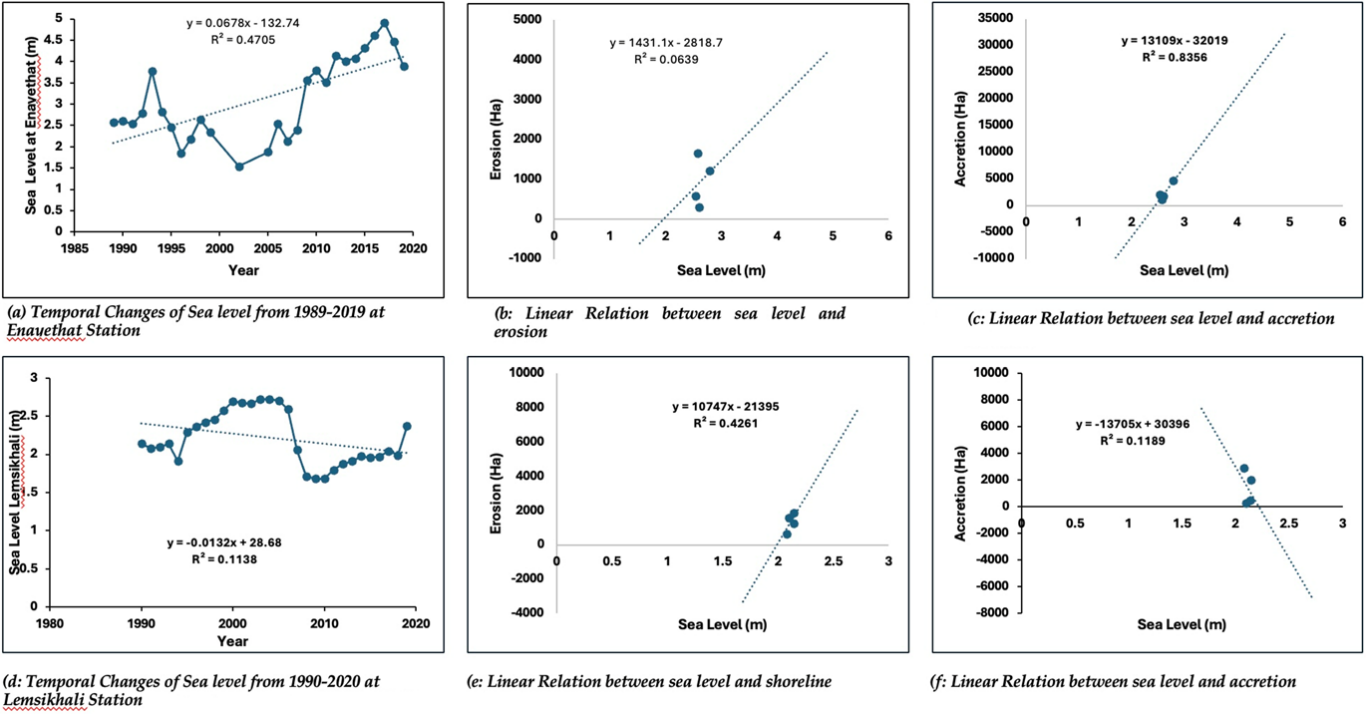
**a** Temporal changes of sea level from 1989 to 2019 at Enayethat Station. **b** Linear relation between sea level and erosion. **c** Linear relation between sea level and accretion. **d** Temporal changes of sea level from 1990 to 2020 at Lemsikhali Station. **e** Linear relation between sea level and shoreline. **f** Linear relation between sea level and accretion

A long and narrow strip of the eastern coast, which is under two administrative districts (Chattogram and Cox’s Bazar), was observed to assess the condition of the erosional-accretional pattern of the shorelines for three decades. These two major districts consist of 11 different upazilas (Table 3). Based on the current study outcome, the total land area along the shoreline was approximately 207.71 thousand ha in 2020. Among these coastal areas, the total accretion recorded for these two districts was 6665.55 m, where 4653.97 m for Chattogram and 2011.58 m for Cox’s Bazar, as well as the total erosion was recorded at 3063.48 m where 1210.50 m was for Chattogram and 1852.98 m for Cox’s Bazar. According to these changes in coastal area statistics, the highest accretion was recorded at 2962.31 m at Mirsarai, Chattogram and the highest erosion was recorded at 1012.54 m at Teknaf, Cox’s Bazar; followed by the lowest accretion was noted at 9.91 m in Anowara, Chattogram and the lowest erosion was noted at 39.14 m in Mirsarai, Chattogram from 1990 to 2020 (Table 2; Fig. 5c). Due to the fluvial geomorphological conditions of the Feni River and the huge sediment accumulation that occurred at this Mirsarai Upazila, Chattogram was the preliminary driver for the highest advancing and lowest retreating shoreline. Conversely, the lowest accretion rate was recorded in Anowara Upazila, Chattogram as depicted in Table 2 and Fig. 4. This was attributed to the low sedimentation rate of the river, as well as significant industrialization along the riverbanks. This occurred despite the area having geomorphological conditions similar to those of Mirsarai. However, the highest erosional pattern was recorded at Teknaf Upazila, Cox’s Bazar, due to the geomorphological conditions of this upazila as this is a geographically narrow part of the south-eastern coastline of Bangladesh where the eastern coast is part of the Bay of Bengal, and the south-western coastline is occupied by the Naf River estuary.

**Table 2:** Erosion and accretion scenarios along the eastern coast of Bangladesh from 1990 to 2020

		** 1990–2000 **		** 2000–2010 **		** 2010–2020 **	
**Districts**	**Upazilas**	**Accretion**	**Erosion**	**Accretion**	**Erosion**	**Accretion**	**Erosion**
Chattogram	Mirsarai	881	83	1212.96	0.24	1044.45	196.62
	Sitakunda	192	410	238.37	132.27	525.28	39.4
	CDA	2	298	75.97	79.95	328.71	74.02
	Anowara	0	189	5.11	14.61	23.27	21.31
	Banshkhali	15	689	245.46	70.78	143.07	250.34
**Chattogram**	**District**	1090	1669	1777.87	297.85	2064.78	581.69
Cox’s Bazar	Chakaria	2	20	10.97	2.9	1.05	36.44
	Maheshkhali	339	796	2436.02	177	106	817.54
	Cox's Bazar	57	29	83.01	15.83	57.85	85.35
	Ramu	27	45	184.81	36.4	41.5	168.91
	Ukhia	15	58	133.87	4.32	27.58	101.1
	Teknaf	29	305	48.59	404.78	52.02	357.93
**Cox’s Bazar**	**District**	2649	4591	6453.01	1236.93	4415.56	2730.65

### Sea-level change

Climate change contributes to rising sea levels, which can cause fluctuations in the shoreline. Additionally, wave action and coastal sedimentation have significantly fluctuating changes in the shoreline’s position over time through erosion and accretion. Adjustment correlation analysis was conducted between low and high tide levels (used as a proxy for sea levels) to better represent the patterns of erosion and accretion processes.

To understand the impact of sea level change along with erosion and accretion, two tidal stations of the Bangladesh Water Development Board (BWDB) were considered in this present study from 1989 to 2019 for Enayethat station, close to Chattogram district, and from 1990 to 2019 for Lemesikhali station, close to Cox’s Bazar district. The daily tidal data were accumulated into monthly tidal data, and the monthly average tidal data were accumulated into yearly tidal data. For Chattogram (Enayethat Station), the highest MSL observed in 2017 was about 4.91 m, while the lowest MSL (Fig. 5a) was found in 2002, which was 1.54 m. Though the MSL value fluctuated, the trend line still showed that the sea level was slowly rising at a rate of 84.369 mm/year from 1989 to 2019 (Fig. 5a). The average value of MSL was 3.12 m. There was no (*R*^2^ = 0.06) (Fig. 5b) correlation between the MSL and erosion, while a significant positive correlation exists between MSL and accretion (*R*^2^ = 0.83) (Fig. 5c). At Cox’s Bazar (Lemsikhali station), the mean sea level has shown a negative trend in recent decades and has decreased at a rate of 6.00 mm/year with a 95% confidence level (Table 3). The highest sea level observed in 2002 was 2.72 m, while the lowest sea level observed in 2009 was 1.681 m. The average MSL was 2.20 m. A moderate positive correlation exists between sea level change and erosion (*R*^2^ = 0.43), and no relation has been found between sea level change and accretion (Fig. 5d, e, and f).

**Table 3:** Sea level trend statistics of tide gauge stations along the eastern coast of Bangladesh; the daily data of the tidal gauge stations were used to calculate the sea level trend

Station name	Data availability	Missing year	Sea level trend (mm/yr)	*p*-value	Kendall-Tau	Level of significance
Enayethat	1989–2019	2000–2001	84.369	0.002	0.44	95%
Lemsikhali	1990–2019	NA	− 6.00	− 0.11	− 0.11	95%

## Discussion

The present study attempted to investigate the impact of sea level changes on shoreline shifting of the eastern coast in Bangladesh (starting from the mouth of the Feni River to Bodormokam point) using sea level data for the station of Cox’s Bazar. To understand the impact of sea level data on the eastern coast, we analyzed Landsat Imagery. The eastern coast of Bangladesh is geologically controlled by the nearest hills. It is a narrow strip where a series of small hills run parallel to the shoreline. The study has been conducted in four segments to understand the shifting patterns and erosion and accretion behaviors in different regions using Landsat Imagery. As segment A was situated at the estuary of the Feni River, the depositional activities were prominent due to the active sedimentation process (0.5 billion tons per year) of the GBM (Ganga Brahmaputra Meghna basin) river system (Kuehl et al., 2005; Rahman et al., 2018; Rashid & Rashid, 2014). The average changes of shoreline progradation towards the sea were 636.26 m, while maximum progradation was up to 3.8 km observed here (refer to Table 2, Fig. 4). The mean net shoreline progradation rate was 21.2 m/year towards the sea. The accretion rate was more active (22.0 m/year) than the erosion rate (2.95 m/year) in segment A (refer to Table 1, Fig. 3). The role of the GBM river system was significant for the seaward movement of the shoreline.

In segment B, two important hilly rivers of Bangladesh reach the Bay of Bengal, so erosion and accretional activities are simultaneously happening, though both rivers travel in a controlled geological environment (Faisal & Hayakawa, 2022). Nevertheless, the erosion rate was dominant (5.6 m/year) as illustrated in Table 2, so the shoreline is eroding towards land. Segment C was the most stable part along the eastern coast, where we found the comparatively low inward movement of the shoreline in the last 30 years. The longest unbroken sea beach in the world, Cox’s Bazar, is situated in this segment. Maheskhali Island is adjacent to the mainland in this portion. The accretion rate (9.79 m/year) (Fig. 5b, Table 1) was comparatively higher than erosion 8.10 m/year A previous study deemed that along the Cox’s Bazar coast, the accretion was more dominant than erosion (Anwar et al., 2022). Only the tail end of the eastern coast of segment D showed some reverse movement, where about 2.89 km (Table 1) of landward movement was recorded, which was the maximum for this segment. Comparing 4 segments, the average erosion rate was dominant in segment D and decreased at a rate of 8.15 m/year. The rising sea level plays a significant role in dominating the erosion pattern of the shoreline (Anwar et al., 2022). The results from the four segments revealed that the accretion rate (21.2 m/year) was more dominant along the eastern coast of Bangladesh compared to the erosion rate (8.15 m/year). The accretion pattern was more dominant when compared to the erosion trends on the eastern coast of Bangladesh. This similar pattern of eastern coast shoreline progradation had been reported previously by Sarwar and Woodroffe (2013) and Matin and Hasan (2021). Due to active sedimentation processes at the estuary of the GBM, the shoreline gained a substantial amount of land (2780.33 ha) from 1990 to 2020, with an annual progression rate of 92.67 ha/year.

In contrast to our regional trend, some regions with similar deltaic environments have exhibited diverse shoreline change patterns. For instance, Besset et al. (2019), Besset et al. (2016), and Gopinath and Seralathan, (2005) reported that in many deltas, erosion rates exceeded those of progradation. Conversely, a more balanced interaction between shoreline erosion and progradation processes (Afolabi & Darby, 2022). Nonetheless, from a global perspective, fluvial deltaic systems have experienced a predominance of erosion over progradation, largely driven by anthropogenic disturbances, such as dam construction, land-use changes, and sediment extraction, particularly since the twentieth century (Besset et al., 2019). The average MSL value (Enayethat station) indicated that the tidal level has been rising over time (1989–2019), while a decreasing trend was observed at Lemsikhali station (1990–2019). Six different graphs were prepared to understand the influence of sea level change. A positive correlation between MSL and shoreline change indicated that the landmass was eroded or going underwater due to the rise of MSL. Despite the rising trend of mean sea level (MSL) at Enayetpur (Fig. 5a), there was minimal or no impact on the eastern coast of Bangladesh, as accretion processes were dominant along the upper coast, particularly in the Chattogram region. In contrast, the MSL at Lemshikhali showed a decreasing trend (Fig. 5d), which was positively associated with increased erosion during the study period. Although the global trend indicates rising sea levels, the abrasion process driven by sediment transport from the GBM system also contributes to the erosion of the landscape along the southern tip of the eastern coast, where the Lemshikhali station is located. Additionally, wind-induced redistribution of energy across the Bay of Bengal and the Indian Ocean plays a crucial role in the seaward shift of the shoreline (Becker et al., 2020; Srinivasu et al., 2017; Thompson et al., 2016), while active sedimentation at the Meghna River estuaries (Kuehl et al., 2005; Rahman et al., 2018; Rashid & Rashid, 2014) significantly contributes to shoreline progradation into the Bay of Bengal. Additionally, GBM river system previously been estimated to vary from 1.0 to 2.4 BT/year which can be separated into components flowing from the Ganges (260 to 680 MT/year) and Brahmaputra (390 to 1160 MT/year) and small contribution of the Meghna system (6–12 MT/year) to the total sediment flux of the GBM system: 150 to 590 MT/year for the Ganges versus 135 to 615 MT/year for the Brahmaputra, with an average total flux around 500 MT/year (Rahman et al., 2018). During the monsoon season (June to October), river flow increases significantly, whereas during the dry season (March to May), water availability is scarce. However, sediment data for the stations within the study area are not available.

The geology of the floodplains, fluvial geomorphological processes, ongoing sedimentation, and deltaic transformations are characteristic features of many active delta systems worldwide. The unique geographical location and geomorphological formations of this coastal system influence the ongoing processes of both progradation and erosion. These natural dynamics are increasingly affected by land-use changes and various forms of anthropogenic interference (Marchesiello et.al., 2019; Bentley et al., 2016; Besset et al., 2017; Zhu et al., 2018). Field observations also confirmed that shoreline progradation has resulted in increased land deposition, which is being utilized by local communities for habitation and livelihood. This trend aligns with a population increase of approximately 98% since 1991, with an average annual growth rate of 2.27% in the region, according to the Bangladesh Bureau of Statistics (2023).

There are some limitations that need to be considered to utilize the findings of this study. One of the constraints of this study is the use of medium-resolution (30 m) Landsat imagery, as finer-scale changes may not be adequately detected. Satellite images were not acquired on the same date, primarily due to cloud cover, which remains a significant challenge in obtaining temporally consistent datasets. This study could not incorporate recent (2024) data, disrupting the decadal time intervals (1990–2000, 2000–2010, 2010–2020), potentially affecting trend analysis and higher temporal scale comparisons. Additionally, shoreline extraction was conducted along the landward margin of the mangrove fringes, which may not fully capture dynamic coastal processes. Another limitation was the inconsistency of tidal level data, with Enayethat station lacking data from 2000 to 2001, which may impact the accuracy of shoreline change assessments. Despite these limitations, the study represents the dynamics of shoreline under current sea-level conditions and the sediment movement processes in GBR. Furthermore, future studies could incorporate the use of RADAR or high-resolution optical imagery to enhance data accuracy and reliability. Additionally, future research should consider the influence of wind energy across the Bay of Bengal and its impacts on shoreline changes.

## Conclusion

Detecting the change of shoreline manual digitization at a fixed zoom scale would help to prepare a better output than automated methods. This study analyzed the impact of rising sea levels along the eastern coast of Bangladesh using multi-dated satellite imageries and topographic maps from 1990 to 2020. A fixed zoom scale (1:2500) was applied to extract the position of the shoreline. A total of 1479 transects were generated by the DSAS tool to study the rate of shoreline change. Among 1479 transects, 41.85% of transects recorded accretion, and 58.14% of transects recorded erosion. The results depicted that the accretion trend dominated along the eastern coast, although the percentage of positive change transects was comparatively lower than negative transects. The maximum accretion area was noticed at Mirsarai (129.04 m/year). The maximum eroded area was noticed along the belt of Teknaf (96.61 m/year). Although sea level is rising along the northeastern coast, a weak uphill positive correlation (*R*^2^ = 0.06) was found, which did not have a significant impact along the northeastern coast of Bangladesh. While sea level is decreasing along the southeastern coast, but a moderate uphill positive correlation was observed during the study period. Therefore, it can be said that sea-level rise does not have any impact on shoreline change. One of the government reports of the Bangladesh General Economics Division (2018) illustrated that no significant impact has been found along the eastern coast despite sea-level rise. This is because the eastern coast of Bangladesh is relatively more stable compared to other parts of the country, where storm surges are less frequent and less impactful. The geological boundary (hills and highlands) and sediment from the rivers would restrict the negative movement of the shoreline. Additionally, sea current is vastly influenced by wind speed and direction, which is a better choice for further study. In response to coastal development and erosion challenges, the Government of Bangladesh has undertaken several strategic initiatives in accreted coastal zones. Particularly, the Mirsarai Economic Zone has been developed, and the Matarbari Thermal Power Station, a major infrastructure project, is currently under implementation in Maheshkhali. Additionally, the construction of the Marine Drive from Cox’s Bazar to Teknaf has been initiated as a protective measure to mitigate the impacts of coastal erosion and enhance regional connectivity. Continuous monitoring is needed to recognize the changes not only on the east coast but also in other coastal regions of Bangladesh. Overall, the results of the study represent significant implications for understanding the dynamic behaviors of open shorelines as well as mangrove-occupied shorelines. These insights are critical for informing adaptive coastal management strategies and enhancing resilience in those countries that are facing the challenges of rising sea levels.

## Data Availability

The research data supporting this study will be made available upon reasonable request.
